# Novel starting points for fragment-based drug design against mycobacterial thioredoxin reductase identified using crystallographic fragment screening

**DOI:** 10.1107/S2059798323005223

**Published:** 2023-08-14

**Authors:** Friederike T. Füsser, Jan Wollenhaupt, Manfred S. Weiss, Daniel Kümmel, Oliver Koch

**Affiliations:** a Institute of Pharmaceutical and Medicinal Chemistry, Münster University, Corrensstrasse 48, 48149 Münster, Germany; b German Center of Infection Research, Münster University, Corrensstrasse 48, 48149 Münster, Germany; c Institute of Biochemistry, Münster University, Corrensstrasse 36, 48149 Münster, Germany; d Macromolecular Crystallography Group, Helmholtz-Zentrum Berlin, Albert-Einstein-Strasse 15, 12489 Berlin, Germany; University of Queensland, Australia

**Keywords:** crystallographic fragment screening, *Mycobacterium tuberculosis*, *Mycobacterium smegmatis*, thioredoxin reductase, antituberculotic drugs

## Abstract

The increasing number of people dying from tuberculosis and the existence of extensively drug-resistant strains has led to an urgent need for new antituberculotic drugs with alternative modes of action. As part of the thioredoxin system, thioredoxin reductase (TrxR) is essential for the survival of *Mycobacterium tuberculosis* (Mtb) and shows substantial differences from human TrxR, making it a promising and most likely selective target. As a model organism for Mtb, crystals of *Mycobacterium smegmatis* TrxR that diffracted to high resolution were used in crystallographic fragment screening to discover binding fragments and new binding sites. The application of the 96 structurally diverse fragments from the F2X-Entry Screen revealed 56 new starting points for fragment-based drug design of new TrxR inhibitors. Over 200 crystal structures were analyzed using *FragMAXapp*, which includes processing and refinement by largely automated software pipelines and hit identification via the multi-data-set analysis approach *PanDDA*. The fragments are bound to 11 binding sites, of which four are positioned at binding pockets or important interaction sites and therefore show high potential for possible inhibition of TrxR.

## Abstract 2

Crystallographic fragment screening of a mycobacterial thioredoxin reductase revealed 56 starting points for the development of new antituberculotic drugs, with 42 fragments bound to 11 different binding sites.

## 1. Introduction

The resurgence of tuberculosis (TB), caused primarily by *Mycobacterium tuberculosis* (Mtb), and the appearance of multidrug-resistant and extensively drug-resistant strains has led to an urgent need for new antituberculotic drugs with alternative modes of action. The World Health Organization’s Tuberculosis Report 2021 showed that TB deaths increased from 2019 (1.4 million) to 2020 (1.5 million), while new infections decreased by 18%. This was explained by the impact of COVID-19 on access to diagnosis and treatment during lockdowns and to both diseases showing similar symptoms. These observations raise concerns that the situation will worsen significantly in the coming years (World Health Organization, 2021 ▸). Approximately 3% of all new infections can be attributed to multidrug-resistant or extensively drug-resistant TB. While the modern treatment of drug-susceptible TB requires a six-month regimen of four first-line drugs, treatment of drug- or multidrug-resistant TB requires long, toxic and expensive treatment, with a high proportion of unsatisfactory results (World Health Organization, 2021 ▸; Diel *et al.*, 2014 ▸; Law *et al.*, 2014 ▸).

Mtb is spread by the inhalation of contaminated aerosol droplets and deposition on the surface of pulmonary alveoli, leading to a high level of oxidative stress for the bacteria during infection and transmission (Hu & Coates, 2009 ▸; Jaeger *et al.*, 2004 ▸; Master *et al.*, 2002 ▸; Smith, 2003 ▸). The mycobacterium depends on a strong oxidative defense system to survive this stress. While Mtb lacks a glutathione system, the bacterium expresses the NADPH-dependent thioredoxin system. Thio­redoxin reductase (TrxR) is part of this thioredoxin system and is an essential factor for mycobacterial thiol redox homeostasis. It has been shown that *in vivo* induced genetic deletion of Mtb-TrxR eradicated Mtb during both the acute and chronic phases of mouse infection. This makes the interaction between TrxR and its substrate thio­redoxin (Trx) a promising new drug target for the treatment of tuberculosis (Lu & Holmgren, 2014 ▸; Lin *et al.*, 2016 ▸). Additionally, mycobacterial TrxR shows substantial differences from human TrxR, which provides the possibility of designing a specific inhibitor with fewer side effects during treatment. The druggability of Mtb-TrxR has already been shown with a class of compounds derived from a docking-based virtual screening approach (Koch *et al.*, 2013 ▸; Koch & Bering, 2019 ▸), which encouraged further investigations.

**Figure 1:**
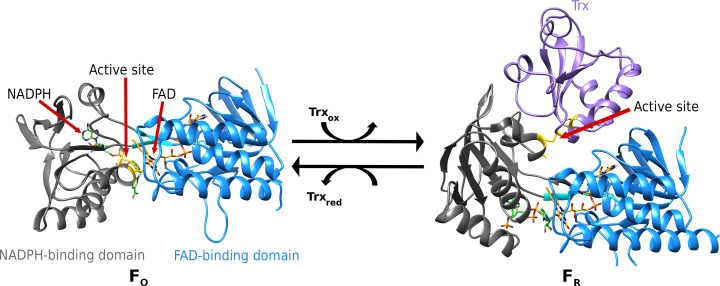
Conformational change of a bacterial TrxR during catalysis. The TrxR can be separated into a NADPH-binding domain (gray) with the cofactor NADPH (green) and a FAD-binding domain (blue) with bound FAD (gold). Prokaryotic TrxR is dependent on a conformational change in which the FAD-binding domain rotates around the NADPH-binding domain, leading to the reduced active site (yellow) being exposed to the surface for interaction with Trx (purple): left, F_O_ (PDB entry 1tde; Waksman *et al.*, 1994 ▸); right, F_R_ (PDB entry 1f6m; Lennon *et al.*, 2000 ▸). Both proteins of the TrxR–Trx complex have one cysteine of the active site mutated to a serine, leading to the formation of a covalently linked disulfide between TrxR and Trx (Waksman *et al.*, 1994 ▸; Lennon *et al.*, 2000 ▸).

TrxR is composed of a NADPH-binding domain and a FAD-binding domain. While FAD is tightly bound to the protein, NADPH acts as an electron donor and is easily exchanged (Fig. 1 ▸). Two distinct subgroups of TrxR exist in higher eukaryotes and in prokaryotes, which exhibit substantial differences in sequence, mechanism and structure (Lu & Holmgren, 2014 ▸; Williams *et al.*, 2000 ▸). Electron transport from NADPH via FAD to an active-site disulfide is conserved in both subgroups. However, while the TrxRs from higher eukaryotes contain a C-terminal active-site motif that interacts with the substrate Trx (Lu & Holmgren, 2014 ▸; Fritz-Wolf *et al.*, 2007 ▸), prokaryotic TrxRs depend on a conformational change between an oxidized (F_O_) and a reduced (F_R_) conformation. In the F_O_ conformation the FAD reduces the active disulfide of TrxR, while the NADPH is replaced. Upon binding of Trx, the NADPH-binding domain rotates around the FAD-binding domain, exposing the disulfide of TrxR on the surface to form the reduced state (F_R_). Thus, Trx is reduced while FAD is simultaneously reduced by NADPH (Lennon *et al.*, 2000 ▸).

Crystallographic fragment screening is a powerful tool to identify potential binding sites and explore the chemical space of the ligands binding to these sites using a variety of small organic molecules called fragments (Davies & Tickle, 2012 ▸; Schiebel *et al.*, 2016 ▸). Even though the fragments are weak binders, their chemical space can be covered efficiently with smaller collections of compounds (Hall *et al.*, 2014 ▸). Here, we present the result of a crystallographic fragment screening on *M. smegmatis* thioredoxin reductase (Msm-TrxR) as a model for Mtb-TrxR. These two proteins show a sequence identity of 77% and high structural similarity, with a root-mean-square deviation (r.m.s.d.) of 0.879 Å (Supplementary Fig. S1). We used crystals of Msm-TrxR with reproducible diffraction to a resolution of around 1.8 Å and the optimized and validated 96-membered F2X-Entry Screen (Wollenhaupt *et al.*, 2020 ▸). The 96 fragments of the F2X-Entry Screen are presented on a 96-well MRC low-profile plate consisting of 12 columns (01–12) and eight rows (A–H). The naming of the individual fragments thus corresponds to the position of the fragment on the plate. The effort for data processing and refinement caused by the large number of crystal data sets was reduced using *FragMAXapp* and included automated software pipelines (Lima *et al.*, 2021 ▸). Using this optimized method, 68 binding events were observed and 42 fragments were found at 11 different binding sites, with some fragments binding to more than one position. Four of these binding sites show potential for inhibitory activity towards TrxR and were therefore further investigated.

## 2. Materials and methods

### 2.1. Protein expression and purification

The vector encoding *M. smegmatis* TrxR (MysmA.00058.a) containing an N-terminal His tag was kindly provided by Seattle Structural Genomics Center for Infectious Disease (https://www.ssgcid.org/). The vector was heterologously overexpressed in *Escherichia coli* BL21(DE3) cells. The cells were grown in TB medium with 100 mg ml^−1^ ampicillin and were incubated at 37°C. After reaching an OD_600_ of 0.8, the culture was cooled on ice for 30 min. Expression was induced by adding 1 m*M* isopropyl β-d-1-thiogalactopyranoside and incubating the culture for 20 h at 16°C. The cells were harvested (4000*g*, 15 min, 4°C) and stored at −80°C until use.

Cell pellets were resuspended in lysis buffer [50 m*M* Tris–HCl, 300 m*M* NaCl, 5%(*v*/*v*) glycerol, 10 m*M* imidazole pH 8.0] mixed with 2.5 µg ml^−1^ DNAse, 500 µg ml^−1^ lysozyme and 5 m*M* Protease Inhibitor Mix (PIC, SERVA, Heidelberg) and were incubated for 30 min. The cells were lysed by sonification (20 min, 10 s on/15 s off, 60% amplitude) and subsequently centrifuged for 45 min at 28 000*g* at 4°C. The supernatant was loaded onto 2 ml Ni–NTA agarose beads pre-equilibrated with lysis buffer. The beads were washed in three steps by increasing the imidazole concentration (10 ml at 10 m*M*, 20 ml at 20 m*M* and 5 ml at 40 m*M*). The protein was eluted with 250 m*M* imidazole.

Size-exclusion chromatography was performed as a second purification step using gel-filtration buffer (10 m*M* Tris pH 8.0). TrxR was purified using an ENrich SEC 650 10 × 300 column on a Bio-Rad NGC Quest chromatography system (Bio-Rad, Berkeley, USA). Fractions containing the protein of interest were concentrated using Amicon filtration units (Millipore) with a 10 kDa molecular-weight cutoff.

### 2.2. Crystallization

*M. smegmatis* TrxR (23 mg ml^−1^) in 10 m*M* Tris pH 8.0 buffer was mixed with 10 m*M* dithiothreitol and incubated for 1 h at 4°C. Crystals were grown in sitting drops by mixing 1 µl protein solution with 1 µl crystallization buffer [2.16 *M* sodium malonate, 10%(*v*/*v*) glycerol pH 6.5] and incubating at 20°C for three days.

### 2.3. Soaking

Soaking was performed using the F2X-Entry Screen (Wollenhaupt *et al.*, 2021 ▸). 0.4 µl soaking solution [2.05 *M* sodium malonate, 9.5%(*v*/*v*) glycerol, 5%(*v*/*v*) DMSO pH 6.5] was added to each well to give a nominal fragment concentration of 100 m*M* and incubated at room temperature for 5 min. A minimum of four crystals were transferred into each drop and soaked for 3 h at 20°C. Two crystals from each condition were harvested and flash-cooled in liquid nitrogen. Additionally, 15 crystals were soaked in soaking solution without any fragments to collect data sets for the apo structure for *PanDDA* analysis.

### 2.4. Data collection

Diffraction experiments were carried out on beamline BL14.1 at the BESSY II synchrotron-radiation source operated by the Helmholtz-Zentrum, Berlin (Mueller *et al.*, 2015 ▸). All diffraction data were recorded at 100 K using a PILATUS 6M detector. The data-collection strategy was optimized based on the characterization of five apo crystals. For each data set, 900 images with an oscillation range of 0.2° and an exposure time of 0.1 s were recorded at full transmission. A total of 207 data sets were collected with a fixed detector distance of 267.1 mm.

### 2.5. Data processing and hit identification

All data sets were processed using *XDSAPP*, *xia*2/*DIALS* and *xia*2/*XDS* (Sparta *et al.*, 2016 ▸; Winter, 2010 ▸; Winter *et al.*, 2022 ▸; Kabsch, 2010 ▸). The crystals belonged to space group *P*3_1_21 and diffracted to a mean resolution of 1.82 Å. The structure of the apo protein was solved using *phenix.phaser* (McCoy *et al.*, 2007 ▸) based on data from the best-diffracting apo crystal and PDB entry 5uth as search model (Seattle Structural Genomics Center for Infectious Disease, unpublished work) and was refined using cycles of iterative model building with *Coot* and refinement with* phenix.refine* (Emsley *et al.*, 2010 ▸; McCoy *et al.*, 2007 ▸), leading to deposition as PDB entry 8cci. This structure was then used as a model for the automated refinement pipelines (*fspipeline* and *DIMPLE*) included in *FragMAXapp* (Lima *et al.*, 2021 ▸; Schiebel *et al.*, 2016 ▸; Agirre *et al.*, 2023 ▸). Hits were identified using *LigandFit* and *PanDDA*, both of which are included in *FragMAXapp* (Pearce *et al.*, 2017 ▸; Venkatachalam *et al.*, 2003 ▸).

## 3. Results and discussion

### 3.1. Crystal optimization and data collection

In order to successfully perform crystallographic fragment screening, reproducible and well diffracting crystals are needed. Therefore, different initial crystal hits for Msm-TrxR were tested and optimized. Optimized crystals were fully grown after three days in crystallization buffer with 10% glycerol and were stable after soaking in buffer containing an additional 5% DMSO for more than 3 h. After the soaking process the crystals showed diffraction to an average resolution of 1.8 Å. All tested crystals belonged to space group *P*3_1_21 and included a single protein molecule per asymmetric unit with the F_O_ conformation of TrxR. Electron density was present for the cofactor FAD and residues 6–310, with only five residues at the N-terminus and one residue at the C-terminus missing. The initial apo structure was solved using the published structure of Msm-TrxR (PDB 5uth; Seattle Structural Genomics Center for Infectious Disease, unpublished work) as a model for molecular replacement at a resolution of 1.56 Å. Our structure (PDB entry 8cci) showed the same conformation as the previously solved structure of Msm-TrxR, consisting of a dimer connected mainly by the α-helices in the FAD-binding domain. Data collection and refinement statistics are summarized in Tables 1 ▸ and 2 ▸.

Optimized crystals and soaking conditions were used to test the 96 structurally diverse fragments of the F2X-Entry Screen (Wollenhaupt *et al.*, 2020 ▸). Two data sets for every fragment and 15 data sets for apo crystals soaked without any fragments were collected. All data sets were collected using the same parameters. Six fragments (depicted in Supplementary Fig. S2) led to a total loss of diffraction and no data could be collected. For the remaining fragments, the resolution of the data sets ranged between 1.1 and 3.5 Å, with a median resolution of 1.8 Å. All data sets were processed and refined using the automated processing pipelines included in *FragMAXapp* and the initial apo crystal structure (PDB entry 8cci).

### 3.2. Fragment screening leading to a hit rate of 44%

**Figure 2:**
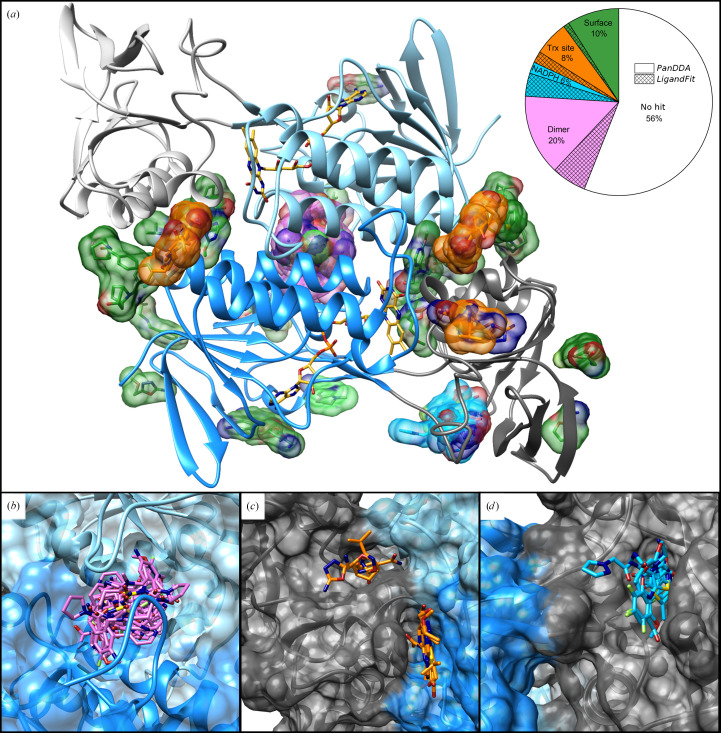
42 fragments bound to 11 binding sites in Msm-TrxR. The diagram depicts the distribution of bound and unbound fragments after *LigandFit* analyses (hatched areas) and after *PanDDa* analyses. All fragments found within *LigandFit* were also found using *PanDDA*. (*a*) Msm-TrxR shown as a dimer (NADPH-binding domain in shades of gray, FAD-binding domain in shades of blue, FAD in yellow). (*b*) 20% of all fragments are bound at the dimer interaction site and are colored pink; before *PanDDA* only six out of 31 fragments were found. (*c*) The Trx interaction site can be separated into two clusters containing 8% of all fragments, shown in orange. Only two fragments were identified by *LigandFit*. (*d*) Nine fragments were found bound to the NADPH-binding pocket and are shown in blue. Four fragments had already been identified as binders by *LigandFit*. Fragments bound to the surface of the protein (10%) are colored green; most of these do not indicate possible inhibition. This leads to hit rates of 13.5% after *LigandFit* and 44% after *PanDDA*.

Initially, the refined data sets were screened for bound fragments using *LigandFit*. A total of 13 fragments were found to bind to four different positions in the protein (see Supplementary Fig. S3), corresponding to a hit rate of 13.5%. A subsequent analysis using *PanDDA* identified 29 additional fragments binding to a total of 11 different binding regions. Interestingly, 14 of these 42 bound fragments were found to bind to more than one region, leading to a total of 68 unique binding events, as shown in Fig. 2 ▸. Overall, an outstanding hit rate of 44% was achieved using the *PanDDA* workflow. 11 fragments were found to bind to seven different binding sites on the surface of TrxR. Seven of these fragments do not bind at the interaction sites of the enzyme and do not show a high potential to inhibit its activity. Therefore, these fragments (shown in Supplementary Fig. S5) will not be further discussed. The four remaining binding sites are located at functionally important interaction sites of the protein. All of the fragments bound to one of these locations show potential to disturb the protein activity. These 56 binding events are possible starting points for fragment-based drug design and their binding sites will be further discussed in the following.

### 3.3. Dimer interaction site

**Figure 3:**
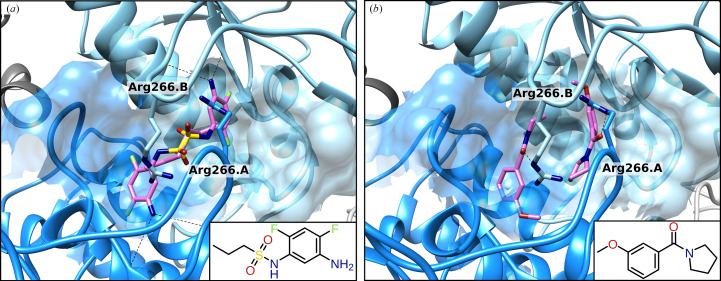
Dimer interaction site of Msm-TrxR with fragments shown in pink. The pocket is located between the FAD-binding domains (shades of blue) of both protomers. All fragments are located twice in the binding pocket, one from each protomer. Fragments D02 (*a*) and D06 (*b*) as examples show that the fragments are mirrored in the binding pocket and interact with both protomers (hydrogen bonds are shown as black dashed lines).

One interesting binding region is located at the dimer interaction site. It represents the largest binding pocket and is well covered with 31 bound fragments (Fig. 2 ▸ *a* and Supplementary Fig. S5). Due to its position at the dimer interface, the binding pocket consists of two identical parts, one from each FAD-binding domain. This leads to two identical fragments in the overall binding site that mirror each other and interact with both protomers. Examples of two bound fragments (D02 and D06) are shown in Fig. 3 ▸. The extensive binding pocket is formed by 60 amino acids and provides various binding positions with different interaction capabilities. The large number and the broad spectrum of different bound fragments show the potential of this binding pocket. All 31 fragments superimposed fill the pocket almost completely. A putative mechanism to inhibit protein activity via the dimer binding pocket may result from the conformational change required for TrxR function. Before TrxR can interact with Trx, the NADPH-binding domain and FAD-binding domain need to rotate with respect to each other. A ligand that stabilizes the F_O_ conformation by connecting the protomers might inhibit the functionality. As a next step to exploit this binding pocket, its dynamics during the conformational change need to be analyzed. With the large number of interacting fragments, it would then be possible to design a ligand that binds with high affinity to both protomers. An interesting method would be to merge or link a fragment bound twice and symmetrically in the binding pocket. This would result in a symmetric ligand that makes the same interactions with both protomers.

### 3.4. Trx-binding site

**Figure 4:**
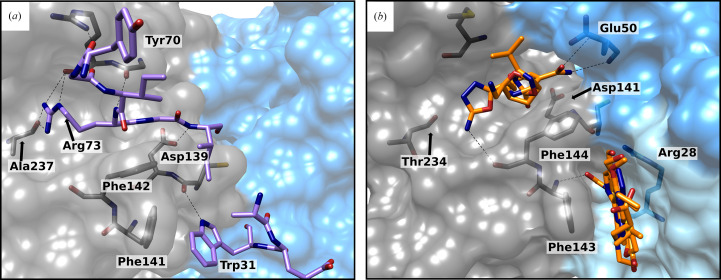
Comparison of the TrxR–Trx complex in *E. coli* with two fragment clusters at the Trx site in Msm-TrxR. (*a*) X-ray structure (PDB entry 1f6m; Lennon *et al.*, 2000 ▸) showing Trx interacting with the F_R_ conformation of *E. coli* TrxR (gray, NADPH-binding domain; blue, FAD-binding domain; purple, Trx). The Trx loop (Tyr70–Ile75; purple) interacts with a binding cleft in the NADPH-binding domain (gray) in the F_R_ conformation. (*b*) Fragments (orange) bound at the Trx interaction site can be separated into two clusters and interact with both domains and protomers (shades of gray, NADPH-binding domain; shades of blue, FAD-binding domain). Hydrogen bonds are shown as black dashed lines.

For functional activity of the Trx system, the interaction between Trx and TrxR is essential for electron transport. The crystal structure of this complex from *E. coli* (PDB entry 1f6m) showed the interactions between these two proteins (Fig. 4 ▸ *a*), which can be transferred to the mycobacterial complex due to their sequence homology. After rotation of the NADPH-binding domain around the FAD-binding domain, Trx can bind to TrxR. While the Msm-Trx loop Ala67–Ile75 (Tyr70–Ile75 in *E. coli*) interacts with a corresponding complementary cleft on the TrxR surface, Phe143 and Phe144 (Phe141 and Phe142 in *E. coli*) interact with a hydrophobic pocket in Trx as described previously (Koch *et al.*, 2013 ▸; Lennon *et al.*, 2000 ▸). If one of these interaction sites is blocked, binding would be inhibited. Thus, fragments bound to these positions are of great interest.

Two clusters of fragments were found to bind at these interaction sites (Fig. 4 ▸ *b* and Supplementary Fig. S6). The first cluster with four fragments binds in the TrxR cleft, where Arg70 (which corresponds to Arg73 in *E. coli*) of Trx interacts with the TrxR backbone of Thr234 (Ala237 in *E. coli*). Since the fragments bind in the F_O_ conformation with a closed Trx-binding site, they also interact with Glu50 in a loop of the FAD-binding domain. While these four fragments do not interact with exactly the same side chains as Arg70, they still block the binding site sterically. Interestingly, all four fragments bind differently in this region and therefore show diverse interactions. Taking the interactions of Arg70 of Trx into account, the fragments can presumably be optimized to ligands that form similar interactions.

The second cluster with seven fragments is located in a pocket between the dimers and is built upon movement of Phe143 and Phe144. All bound fragments include an aromatic ring and are aligned with Phe143, showing π–π stacking. Phe144 frames the binding site. The surrounding polar side chains (Glu52*A*, Arg28*B*, Arg76*B* and Gln30*B*) and aromatic side chains (Phe143*A*, Phe144*A*, Phe169*A* and Phe77*B*) represent a large number of potential interaction partners for a wide range of different fragments. Therefore, fragments could be optimized to interact with both protein protomers, which might stabilize the binding of the dimer. Another interesting step would be to connect both clusters by a bridge. This could help to generate a highly potent compound that inhibits the interaction with Trx in two ways.

### 3.5. NADPH-binding pocket

**Figure 5:**
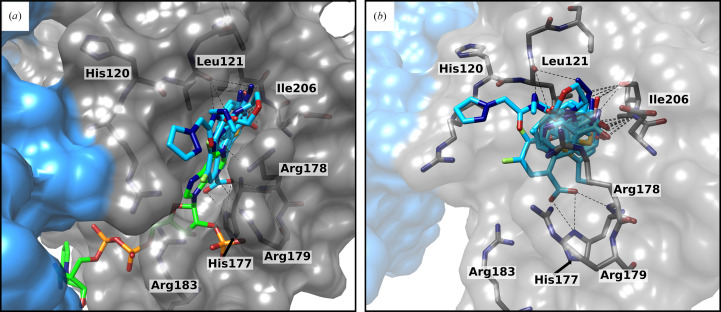
Close-up view of the NADPH-binding pocket of Msm-TrxR with bound fragments (light blue) in comparison to bound NADPH (green). (*a*) In comparison to the bound NADPH (green, overlay from PDB entry 8ccj), the bound fragments are located deeper in the binding site. Arg178, Arg179 and Arg183 show rearrangement around the fragments and close the binding site in comparison to the NADPH-bound conformation. (*b*) Nine fragments are bound to the NADPH-binding pockets and show hydrogen bonds to the backbone of Leu121, Ile206 and His177. Hydrogen bonds are shown as black dashed lines.

The enzymatic activity of thioredoxin reductase is dependent on the supply of electrons by the cofactor NADPH. Therefore, the NADPH-binding pocket is also of interest for the design of bioactive inhibitors. Unfortunately, a large number of NADPH-binding proteins exist and inhibitors need to be selective for the mycobacterial TrxR pocket. This fragment screening revealed nine fragments that bind in the NADPH-binding pocket (Fig. 5 ▸ and Supplementary Fig. S7) and also reach into pockets outside the NADPH-binding site. Hydrogen bonds to His177 and the backbone of Leu121 are observed, but most of the fragments form hydrogen bonds to the backbone of Ile206. Due to fragment binding, Arg183 and Arg178 seem to be more ordered in the binding pocket and show slight movements compared with the NADPH-binding conformation. These movements lead to a closing of the binding pocket, with Arg183 and Arg178 framing the binding site. All bound fragments include an aromatic ring system (Supplementary Fig. S7). A comparison to the structure with bound NADPH shows that the fragments reach deeper inside the binding site and therefore extend the actual NADPH-binding pocket. Four fragments had already been found in the *LigandFit* analysis, which hints at higher occupancy of the fragment in the crystal, which in turn is generally loosely correlated with higher affinity. If fragments could be optimized to bind specifically in the NADPH-binding pocket of bacterial or mycobacterial TrxR, this could be a great starting point for a new class of antituberculotic drugs.

### 3.6. Interaction site of the NADPH- and FAD-binding domains

**Figure 6:**
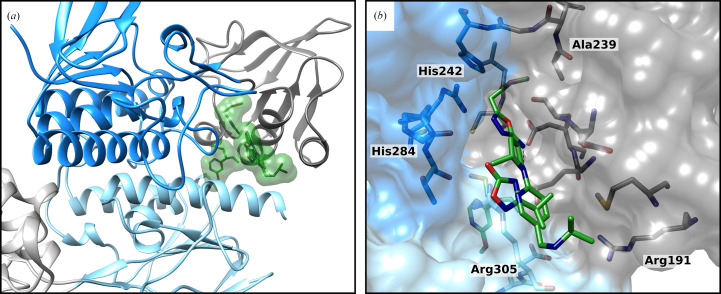
Single fragments bound to the surface between the FAD-binding and NADPH-binding domains. (*a*) Overall structure of four fragments (green) bound between the NADPH-binding domain (gray) and the FAD-binding domain (blue). (*b*) Close-up view of the bound fragments in the interaction site. Hydrogen bonds are shown as black dashed lines.

A subset of fragments are located between the NADPH- and FAD-binding domains of TrxR (Fig. 6 ▸ and Supplementary Fig. S4). This region changes when both domains rotate during the conformational transition between the F_O_ and F_R_ states. In contrast to the other binding sites, which were defined as clusters, these four fragments do not occupy the same position in the interaction site. All four fragments together occupy a significant part of the domain interaction site. A molecule that binds here with high affinity could sterically block the rotation of the domains with respect to each other. With this movement blocked, TrxR can no longer perform its enzymatic activity and would be inhibited. For further investigations, it will be interesting to test additional fragments, with the hope of forming a cluster at this interaction site and gaining more information.

## 4. Conclusion

This crystallographic fragment screening revealed interaction sites for 42 out of 96 structurally diverse fragments from the F2X-Entry Screen in Msm-TrxR after soaking the fragments into optimized protein crystals that diffract to high resolution. The initial hit rate of 13 fragments identified using *LigandFit* was tripled using *PanDDA* analyses. 41 fragments were found in four interesting binding regions with the potential to inhibit TrxR activity. Since some fragments were found in more than one binding region, a total of 56 starting points for fragment-based drug design are now available. PanDDA analysis data are publicly available from Münster University data storage. The PanDDA results are split into two datasets due to their size (https://doi.org/10.17879/40019550156 and https://doi.org/10.17879/69998577695). The data are described in Table S3 in the Supporting Information. In addition, the resulting pdb files can be downloaded from https://doi.org/10.17879/39998753552.

## Supplementary Material

PDB reference: *Mycobacterium smegmatis* thioredoxin reductase, 8cci

Supporting Information. DOI: 10.1107/S2059798323005223/jb5056sup1.pdf

PanDDA results Dataset 1: https://doi.org/10.17879/40019550156

PanDDA results Dataset 2: https://doi.org/10.17879/69998577695

PanDDA results Dataset 3: https://doi.org/10.17879/39998753552

## Figures and Tables

**Table 1:** Data collection and processing for PDB entry 8cci Values in parentheses are for the highest resolution shell.

Diffraction source	Beamline 14.1, BESSY
Wavelength (Å)	0.918
Temperature (K)	100
Detector	PILATUS
Crystal-to-detector distance (mm)	267.3
Total rotation range (°)	180
Rotation per image (°)	0.2
Exposure time per image (s)	0.1
Space group	*P*3_1_21
*a*, *b*, *c* (Å)	67.79, 67.79, 155.11
α β, γ (°)	90, 90, 120
Mosaicity (°)	0.11
Resolution range (Å)	46.81–1.56 (1.62–1.56)
Total No. of reflections	587455 (95036)
No. of unique reflections	59648 (5883)
Completeness (%)	99.7 (99.6)
Multiplicity	9.8
〈*I*/σ(*I*)〉 from merged data	12.0 (0.5)
CC_1/2_	0.99 (0.5)
*R*_meas_ (%)	13.2 (362.4)
Overall *B* factor from Wilson plot (Å)	24.1

**Table 2:** Structure refinement for PDB entry 8cci Values in parentheses are for the highest resolution shell.

Resolution range (Å)	46.81–1.56
Completeness (%)	99.7
No. of reflections, working set	59520 (5867)
No. of reflections, test set	2094 (207)
Final *R*_cryst_	0.2095 (0.4933)
Final *R*_free_	0.2354 (0.5290)
No. of non-H atoms	
Protein/nucleic acid	2337/305
Ions	1
Ligands	86
Waters	170
Total	2594
R.m.s. deviations from ideality	
Bond lengths (Å)	0.013
Angles (°)	1.11
Average *B* factors (Å^2^)	
Overall	37.30
Protein/nucleic acid	37.16
Ions	36.03
Ligands	32.29
Waters	41.41
Ramachandran plot	
Favored regions (%)	98.0
Outliers (%)	0.3
Unmodelled residues (%)	4.3
