# Supplementary material for: Novel starting points for fragment-based drug design against mycobacterial thioredoxin reductase identified using crystallographic fragment screening
Source: Acta Crystallogr D Struct Biol. 2023 Aug 14;79(Pt 9):857–65. doi: 10.1107/S2059798323005223 (PMC10478635; doi:10.1107/S2059798323005223)
Supplement: Supplementary file 1 [file d-79-00857-sup1.pdf]

STRUCTURAL
BIOLOGY

Volume 79 (2023)

Supporting information for article:

Novel starting points for fragment-based drug design against mycobacterial thioredoxin reductase identified using crystallographic fragment screening

Friederike T. Füsser, Jan Wollenhaupt, Manfred S. Weiss, Daniel Kümmel and Oliver Koch

A

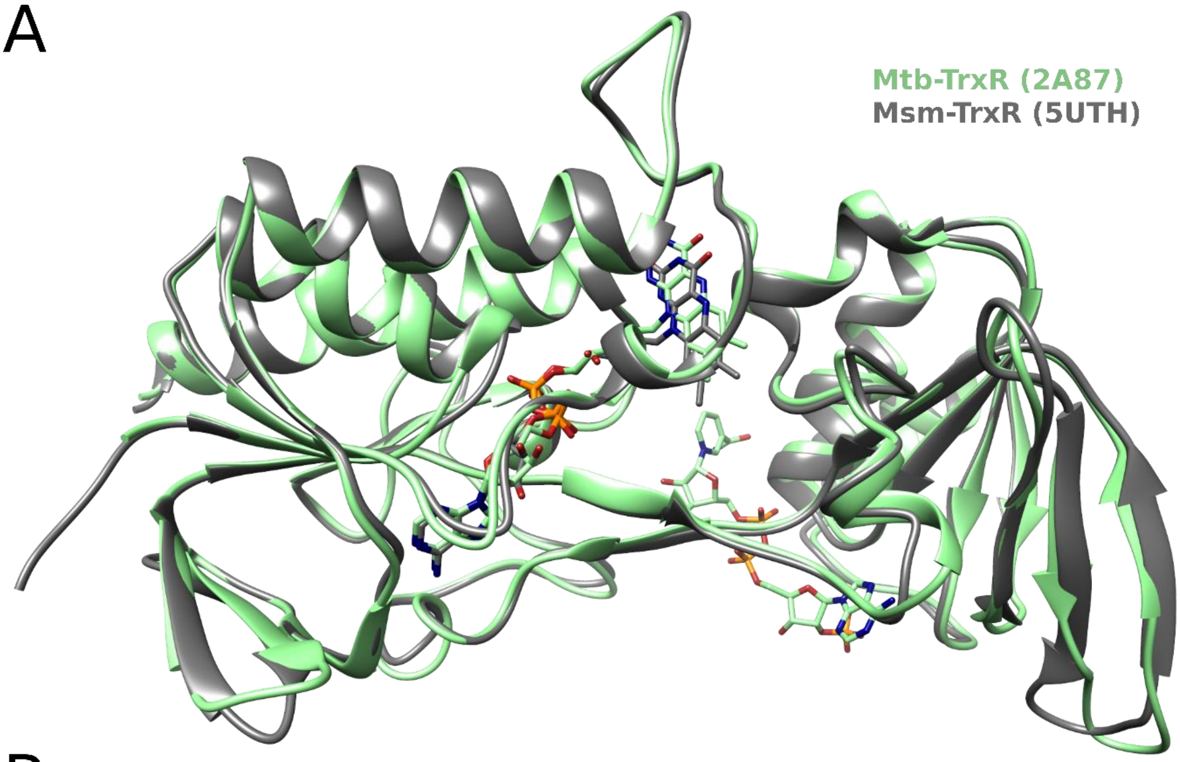

B

```
Mtb-TrxR      MTAPPVHDLAHHPVRDVIVIGSGPAGYTAALYAARAQLAPLVFEGTSFGGALMTTTDVEN 60
Msm-TrxR      -------MSTSQTVHDVIIIGSGPAGYTAAIYAARAQLKPLVFEGTQFGGALMTTTEVEN 53
                       : : *:***:***********:******* *******.*********:***

Mtb-TrxR      YPGFRNGITGPELMDEMREQALRFGADLRMEDVESVSLHGPLKSVVTADGQTHRARAVIL 120
Msm-TrxR      YPGFREGITGPELMDQMREQALRFGADLRMEDVDAVQLEGPVKTVVVGD-ETHQARAVIL 112
              *****:*********:*****************::*.*.**:*:**..* :**:******

Mtb-TrxR      AMGAAARYLQVPGEQELLGRGVSSCATCDGFFFRDQDIAVIGGGDSAMEEATFLTRFARS 180
Msm-TrxR      AMGAAARHLGVPGEEALTGMGVSTCATCDGFFFRDQDIVVVGGGDSAMEEATFLTRFARS 172
              *******:* ****: * * ***:**************.*:*******************

Mtb-TrxR      VTLVHRRDEFRASKIMLDRARNNDKIRFLTNHTVVAVDGDTTVTGLRVRDTNTGAETTLP 240
Msm-TrxR      VTLIHRRDEFRASKIMLERARANEKITFLTNTEITQIEGDPKVTGVRLRDTVTGEESKLD 232
              ***:*************:*** *:** ****  :. ::** .***:*:*** ** *:.* 

Mtb-TrxR      VTGVFVAIGHEPRSGLVREAIDVDPDGYVLVQGRTTSTSLPGVFAAGDLVDRTYRQAVTA 300
Msm-TrxR      VTGVFVAIGHDPRSELVRGQVELDDEGYVKVQGRTTYTSLDGVFAAGDLVDHTYRQAITA 292
              **********:*** ***  :::* :*** ****** *** **********:*****:**

Mtb-TrxR      AGSGCAAAIDAERWLAEHAATGEADSTDALIGAQR 335
Msm-TrxR      AGSGCAASIDAERWLAEQD---------------- 311
```

Figure S1 Comparison of mycobacterial TrxR. **(A)** The Structural comparison of Mtb-TrxR (PDB 2A87, green) and Msm-TrxR (5UTH, gray) lead to an RMSD of 0.879 Å using MOE. **(B)** A sequence alignment using clustal omega showed a sequence identity of 77%.

Table S1 Macromolecule production information for PDB 8CCI

Source organism	Mycobacterium smegmatis MC2 155
DNA source	
Expression vector	BG1816
Plasmid construction method	*Provided by SSGCID*
Expression host	Escherichia coli BL21(DE3)
Expression details	Tb-medium, induced by 1 mM IPTG, 16 hours expression at 16°C
Complete amino-acid sequence of the protein	MAHHHHHHMSTSQTVHDVIIIGSGPAGYTAAIYAAR AQLKPLVFEGTQFGGALMTTTEVENYPGFREGITGPE LMDQMREQALRFGADLRMEDVDAVQLEGPVKTVV VGDETHQARAVILAMGAAARHLGVPGEEALTGMGV STCATCDGFFFRDQDIVVVGGGDSAMEEATFLTRFA RSVTLIHRRDEFRASKIMLERARANEKITFLTNTEITQI EGDPKVTGVRLRDTVTGEESKLDVTGVFVAIGHDPR SELVRGQVELDDEGYVKVQGRTTYTSLDGVFAAGDL VDHTYRQAITAAGSGCAASIDAERWLAEQD-

Table S2 Crystallization data for PDB 8CCI

Method	Vapor diffusion, sitting drop
Plate type	48 well MRC Maxi Plate (Jena Bioscience)
Temperature (°C)	20
Protein concentration	23 mg mL^{-1}
Buffer composition of protein solution	10 mM Tris at pH 8.0, 10 mM DTT
Composition of reservoir solution	2.16 M Sodium malonate dibasic monohydrate, 10% glycerol
Volume and ratio of drop	2 µL 1:1
Volume of reservoir	100 µL
Composition of the cryoprotectant	2.16 M Sodium malonate dibasic monohydrate, 10% glycerol, 5% DMSO
Drop setting	Specify if manual
Seeding	no

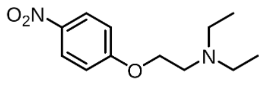

H01

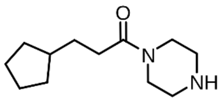

H02

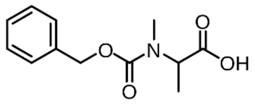

H03

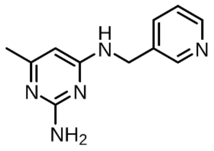

H05

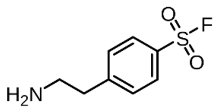

F11

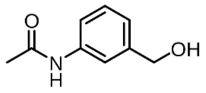

G11

Figure S2 Depicted fragments lead to loss of diffraction after soaking for 3 hours on Msm-TrxR crystals. Fragment F11 contains a warhead that could lead to a covalent binding.

Figure S3 Fragment found to bind after LigandFit analyses in their binding pocket. Corresponding electron density maps (2Fo-DFc) are contoured at σ = 1 (blue) and (Fo-DFc)-maps are contoured at σ = 3 (green).

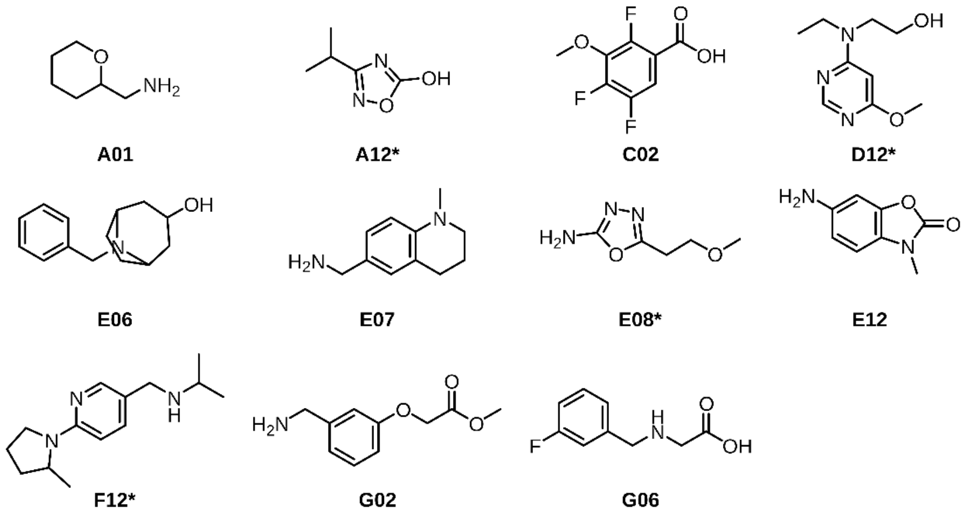

Figure S4 Fragments found binding to the surface of Msm-TrxR. Four fragments, bound between the NADPH- and FAD-domain are marked with (*).

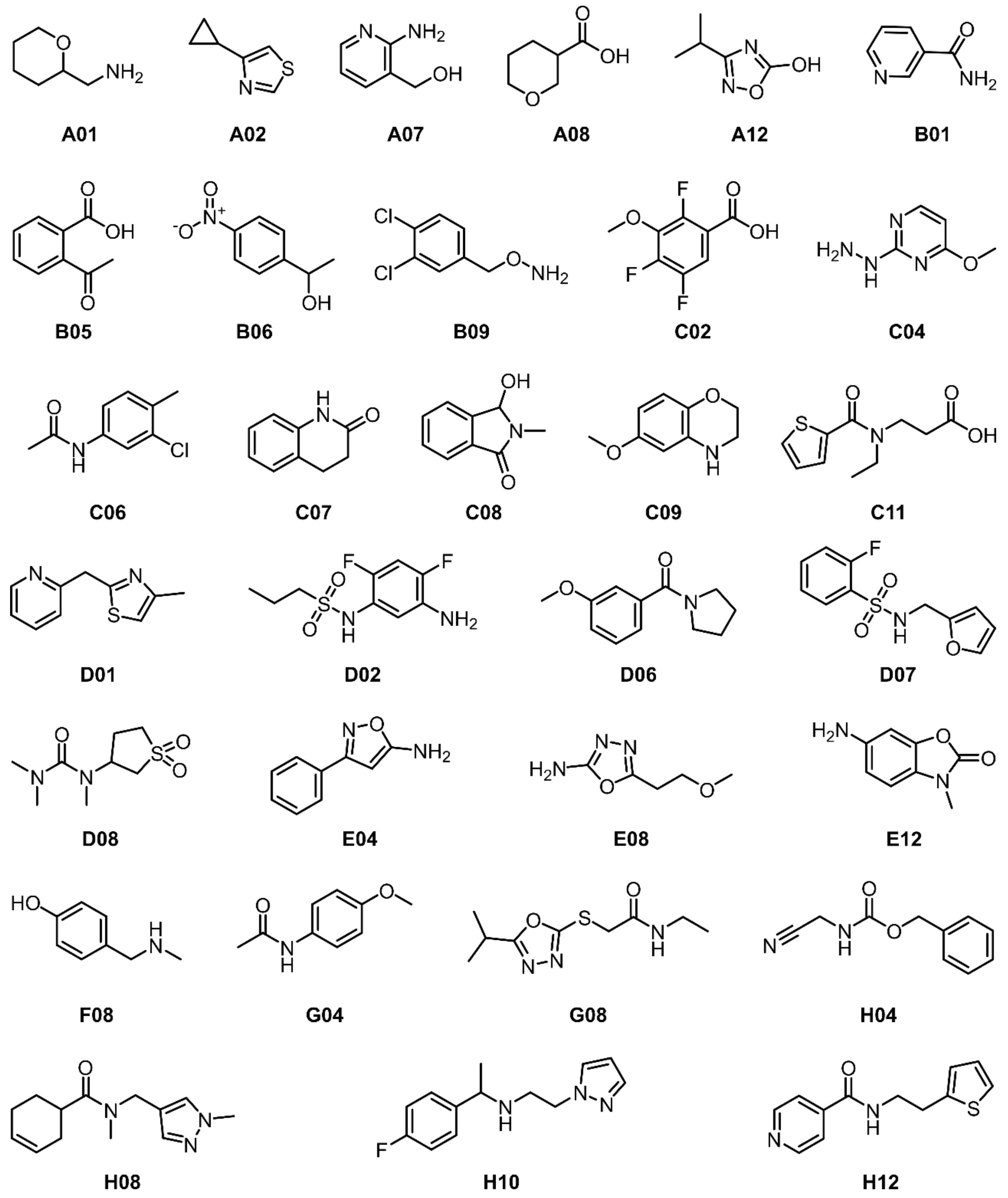

Figure S5 Fragments found binding to the dimer site of Msm-TrxR.

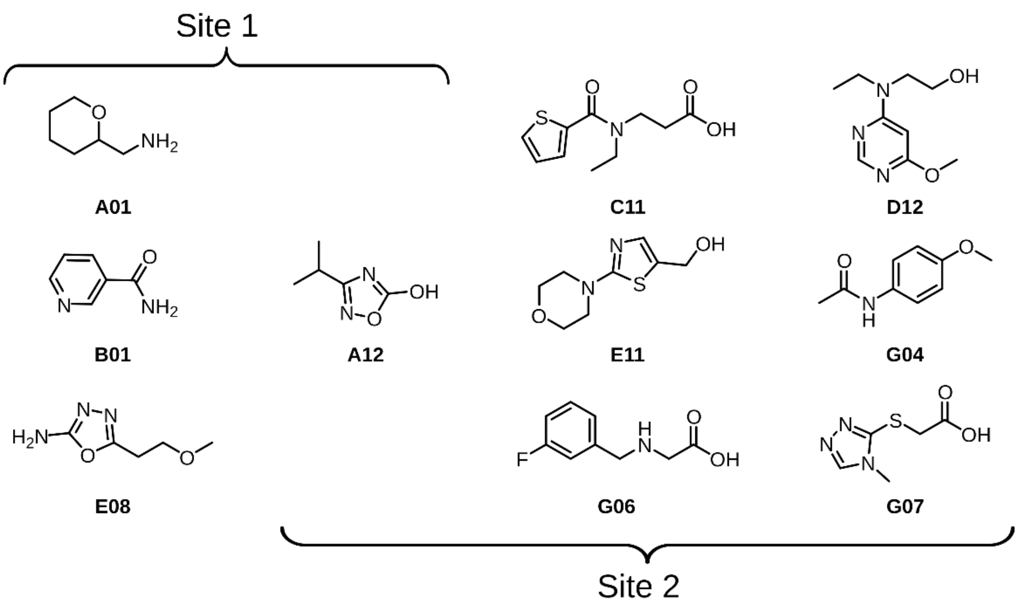

Figure S6 Depicted fragments bound to Trx binding site. Fragments A01, B01, and E08 bind to the first site, A12 can be found in both clusters, while C11, D12, E06, E11, G04, and G07 bind in the second part of the binding pocket.

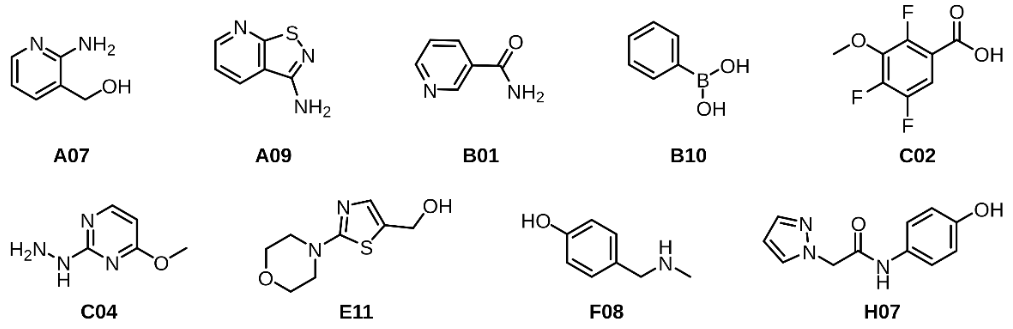

Figure S7 Fragments found binding to the NADPH binding site

Table S3 Available PanDDA results. The datasets can be downloaded from the mentioned DOI.

Fragment	Crystal	Binding site	Processing program	Refinement pipeline	DOI	Data Path to corresponding PDB file
A01	a	Dimer<br>Surface<br>Surface	xdsapp	plex	10.17879/69998577695	FF-PanDDA-Daten2/xdsapp_plex/pandda/processed_datasets/FF-F2XEntry-A01a_1/modelled_structures/FF-F2XEntry-A01a_1-pandda-model.pdb
A01	a	Trx1	xdsapp	dimple	10.17879/69998577695	FF-PanDDA-Daten2/xdsapp_dimple/pandda/processed_datasets/FF-F2XEntry-A01a_1/modelled_structures/FF-F2XEntry-A01a_1-pandda-model.pdb
A02	a	Dimer	frag	plex	10.17879/69998577695	FF-PanDDA-Daten2/frag_plex/pandda/processed_datasets/FF-F2XEntry-A02a_1/modelled_structures/FF-F2XEntry-A02a_1-pandda-model.pdb
A07	b	Dimer<br>NADPH	frag	plex	10.17879/69998577695	FF-PanDDA-Daten2/frag_plex/pandda/processed_datasets/FF-F2XEntry-A07b_1/modelled_structures/FF-F2XEntry-A07b_1-pandda-model.pdb
A08	b	Dimer	xdsapp	dimple	10.17879/69998577695	FF-PanDDA-Daten2/xdsapp_dimple/pandda/processed_datasets/FF-F2XEntry-A08b_1/modelled_structures/FF-F2XEntry-A08b_1-pandda-model.pdb
A09	b	NADPH	frag	plex	10.17879/69998577695	FF-PanDDA-Daten2/frag_plex/pandda/processed_datasets/FF-F2XEntry-A09b_1/modelled_structures/FF-F2XEntry-A09b_1-pandda-model.pdb
A12	b	Dimer	frag	plex	10.17879/69998577695	FF-PanDDA-Daten2/frag_plex/pandda/processed_datasets/FF-F2XEntry-A12b_1/modelled_structures/FF-F2XEntry-A12b_1-pandda-model.pdb
A12	a	Trx1	frag	plex	10.17879/69998577695	FF-PanDDA-Daten2/frag_plex/pandda/processed_datasets/FF-F2XEntry-A12a_1/modelled_structures/FF-F2XEntry-A12a_1-pandda-model.pdb
A12	a	Trx2	xdsapp	dimple	10.17879/69998577695	FF-PanDDA-Daten2/xdsapp_dimple/pandda/processed_datasets/FF-F2XEntry-A12a_1/modelled_structures/FF-F2XEntry-A12a_1-pandda-model.pdb
A12	b	Surface	xdsapp	plex	10.17879/69998577695	FF-PanDDA-Daten2/xdsapp_plex/pandda/processed_datasets/FF-F2XEntry-A12b_1/modelled_structures/FF-F2XEntry-A12b_1-pandda-model.pdb

A12	b	Surface	xdsapp	dimple	10.17879/69998577695	FF-PanDDA-Daten2/xdsapp_dimple/pandda/processed_datasets/FF-F2XEntry-A12b_1/modelled_structures/FF-F2XEntry-A12b_1-pandda-model.pdb
B01	a	Dimer	xdsapp	plex	10.17879/69998577695	FF-PanDDA-Daten2/xdsapp_plex/pandda/processed_datasets/FF-F2XEntry-B01a_1/modelled_structures/FF-F2XEntry-B01a_1-pandda-model.pdb
B01	a	NADPH Trx1	dials	dimple	10.17879/40019550156	FF-PanDDA-Daten1/dials_dimple/pandda/processed_datasets/FF-F2XEntry-B01a_1/modelled_structures/FF-F2XEntry-B01a_1-pandda-model.pdb
B05	b	Dimer	dials	plex	10.17879/40019550156	FF-PanDDA-Daten1/dials_plex/pandda/processed_datasets/FF-F2XEntry-B05b_1/modelled_structures/FF-F2XEntry-B05b_1-pandda-model.pdb
B06	b	Dimer	frag	plex	10.17879/69998577695	FF-PanDDA-Daten2/frag_plex/pandda/processed_datasets/FF-F2XEntry-B06b_1/modelled_structures/FF-F2XEntry-B06b_1-pandda-model.pdb
B09	b	Dimer	xdsapp	plex	10.17879/69998577695	FF-PanDDA-Daten2/xdsapp_plex/pandda/processed_datasets/FF-F2XEntry-B09b_1/modelled_structures/FF-F2XEntry-B09b_1-pandda-model.pdb
B10	a	NADPH	frag	plex	10.17879/69998577695	FF-PanDDA-Daten2/frag_plex/pandda/processed_datasets/FF-F2XEntry-B10a_1/modelled_structures/FF-F2XEntry-B10a_1-pandda-model.pdb
C02	a	Dimer	xdsapp	dimple	10.17879/69998577695	FF-PanDDA-Daten2/xdsapp_dimple/pandda/processed_datasets/FF-F2XEntry-C02a_1/modelled_structures/FF-F2XEntry-C02a_1-pandda-model.pdb
C02	a	NADPH	dials	dimple	10.17879/40019550156	FF-PanDDA-Daten1/dials_dimple/pandda/processed_datasets/FF-F2XEntry-C02a_1/modelled_structures/FF-F2XEntry-C02a_1-pandda-model.pdb
C02	a	Surface	xdsapp	dimple	10.17879/69998577695	FF-PanDDA-Daten2/xdsapp_dimple/pandda/processed_datasets/FF-F2XEntry-C02a_1/modelled_structures/FF-F2XEntry-C02a_1-pandda-model.pdb
C04	b	Dimer	xdsapp	dimple	10.17879/69998577695	FF-PanDDA-Daten2/xdsapp_dimple/pandda/processed_datasets/FF-F2XEntry-C04b_1/modelled_structures/FF-F2XEntry-C04b_1-pandda-model.pdb

C04	b	NADPH	frag	plex	10.17879/69998577695	FF-PanDDA-Daten2/frag_plex/pandda/processed_datasets/FF-F2XEntry-C04b_1/modelled_structures/FF-F2XEntry-C04b_1-pandda-model.pdb
C06	b	Dimer	dials	dimple	10.17879/40019550156	FF-PanDDA-Daten1/dials_dimple/pandda/processed_datasets/FF-F2XEntry-C06b_1/modelled_structures/FF-F2XEntry-C06b_1-pandda-model.pdb
C07	a	Dimer	xdsapp	dimple	10.17879/69998577695	FF-PanDDA-Daten2/xdsapp_dimple/pandda/processed_datasets/FF-F2XEntry-C07a_1/modelled_structures/FF-F2XEntry-C07a_1-pandda-model.pdb
C08	b	Dimer	xdsapp	dimple	10.17879/69998577695	FF-PanDDA-Daten2/xdsapp_dimple/pandda/processed_datasets/FF-F2XEntry-C08b_1/modelled_structures/FF-F2XEntry-C08b_1-pandda-model.pdb
C09	a	Dimer	frag	plex	10.17879/69998577695	FF-PanDDA-Daten2/frag_plex/pandda/processed_datasets/FF-F2XEntry-C09a_1/modelled_structures/FF-F2XEntry-C09a_1-pandda-model.pdb
C11	a	Dimer	dials	fspipeline	10.17879/40019550156	FF-PanDDA-Daten1/dials_fspipeline/pandda/processed_datasets/FF-F2XEntry-C11a_1/modelled_structures/FF-F2XEntry-C11a_1-pandda-model.pdb
C11	b	Surface Trx2	frag	plex	10.17879/69998577695	FF-PanDDA-Daten2/frag_plex/pandda/processed_datasets/FF-F2XEntry-C11b_1/modelled_structures/FF-F2XEntry-C11b_1-pandda-model.pdb
D01	b	Dimer	frag	plex	10.17879/69998577695	FF-PanDDA-Daten2/frag_plex/pandda/processed_datasets/FF-F2XEntry-D01b_1/modelled_structures/FF-F2XEntry-D01b_1-pandda-model.pdb
D02	b	Dimer	frag	plex	10.17879/69998577695	FF-PanDDA-Daten2/frag_plex/pandda/processed_datasets/FF-F2XEntry-D02b_1/modelled_structures/FF-F2XEntry-D02b_1-pandda-model.pdb
D06	a	Dimer	frag	plex	10.17879/69998577695	FF-PanDDA-Daten2/frag_plex/pandda/processed_datasets/FF-F2XEntry-D06a_1/modelled_structures/FF-F2XEntry-D06a_1-pandda-model.pdb
D07	a	Dimer	frag	plex	10.17879/69998577695	FF-PanDDA-Daten2/frag_plex/pandda/processed_datasets/FF-F2XEntry-D07a_1/modelled_structures/FF-F2XEntry-D07a_1-pandda-model.pdb

D08	b	Dimer	frag	plex	10.17879/69998577695	FF-PanDDA-Daten2/frag_plex/pandda/processed_datasets/FF-F2XEntry-D08b_1/modelled_structures/FF-F2XEntry-D08b_1-pandda-model.pdb
D12	a	Surface<br>Trx2	xdsapp	dimple	10.17879/69998577695	FF-PanDDA-Daten2/xdsapp_dimple/pandda/processed_datasets/FF-F2XEntry-D12a_1/modelled_structures/FF-F2XEntry-D12a_1-pandda-model.pdb
D12	b	Surface	dials	dimple	10.17879/40019550156	FF-PanDDA-Daten1/dials_dimple/pandda/processed_datasets/FF-F2XEntry-D12b_1/modelled_structures/FF-F2XEntry-D12b_1-pandda-model.pdb
E04	b	Dimer	xdsapp	dimple	10.17879/69998577695	FF-PanDDA-Daten2/xdsapp_dimple/pandda/processed_datasets/FF-F2XEntry-E04b_1/modelled_structures/FF-F2XEntry-E04b_1-pandda-model.pdb
E06	b	Surface	xdsapp	plex	10.17879/69998577695	FF-PanDDA-Daten2/xdsapp_plex/pandda/processed_datasets/FF-F2XEntry-E06b_1/modelled_structures/FF-F2XEntry-E06b_1-pandda-model.pdb
E06	b	Surface	xdsapp	plex	10.17879/69998577695	FF-PanDDA-Daten2/xdsapp_plex/pandda/processed_datasets/FF-F2XEntry-E06b_1/modelled_structures/FF-F2XEntry-E06b_1-pandda-model.pdb
E07	b	Surface	xdsapp	dimple	10.17879/69998577695	FF-PanDDA-Daten2/xdsapp_dimple/pandda/processed_datasets/FF-F2XEntry-E07b_1/modelled_structures/FF-F2XEntry-E07b_1-pandda-model.pdb
E08	b	Dimer<br>Trx1<br>Surface<br>Surface	xdsapp	dimple	10.17879/69998577695	FF-PanDDA-Daten2/xdsapp_dimple/pandda/processed_datasets/FF-F2XEntry-E08b_1/modelled_structures/FF-F2XEntry-E08b_1-pandda-model.pdb
E11	a	NADPH	frag	plex	10.17879/69998577695	FF-PanDDA-Daten2/frag_plex/pandda/processed_datasets/FF-F2XEntry-E11a_1/modelled_structures/FF-F2XEntry-E11a_1-pandda-model.pdb
E11	b	Trx2	xdsapp	dimple	10.17879/69998577695	FF-PanDDA-Daten2/xdsapp_dimple/pandda/processed_datasets/FF-F2XEntry-E11b_1/modelled_structures/FF-F2XEntry-E11b_1-pandda-model.pdb

E12	b	Dimer<br>Surface	xdsapp	dimple	10.17879/69998577695	FF-PanDDA-Daten2/xdsapp_dimple/pandda/processed_datasets/FF-F2XEntry-E12b_1/modelled_structures/FF-F2XEntry-E12b_1-pandda-model.pdb
F08	b	Dimer	dials	dimple	10.17879/40019550156	FF-PanDDA-Daten1/dials_dimple/pandda/processed_datasets/FF-F2XEntry-F08b_1/modelled_structures/FF-F2XEntry-F08b_1-pandda-model.pdb
F08	b	NADPH	xdsapp	plex	10.17879/69998577695	FF-PanDDA-Daten2/xdsapp_plex/pandda/processed_datasets/FF-F2XEntry-F08b_1/modelled_structures/FF-F2XEntry-F08b_1-pandda-model.pdb
F12	a	Surface	xdsapp	plex	10.17879/69998577695	FF-PanDDA-Daten2/xdsapp_plex/pandda/processed_datasets/FF-F2XEntry-F12a_1/modelled_structures/FF-F2XEntry-F12a_1-pandda-model.pdb
G02	a	Surface	xdsapp	dimple	10.17879/69998577695	FF-PanDDA-Daten2/xdsapp_dimple/pandda/processed_datasets/FF-F2XEntry-G02a_1/modelled_structures/FF-F2XEntry-G02a_1-pandda-model.pdb
G04	b	Dimer	frag	plex	10.17879/69998577695	FF-PanDDA-Daten2/frag_plex/pandda/processed_datasets/FF-F2XEntry-G04b_1/modelled_structures/FF-F2XEntry-G04b_1-pandda-model.pdb
G04	b	Trx2	dials	plex	10.17879/40019550156	FF-PanDDA-Daten1/dials_plex/pandda/processed_datasets/FF-F2XEntry-G04b_1/modelled_structures/FF-F2XEntry-G04b_1-pandda-model.pdb
G06	a	Surface<br>Surface	xdsapp	dimple	10.17879/69998577695	FF-PanDDA-Daten2/xdsapp_dimple/pandda/processed_datasets/FF-F2XEntry-G06a_1/modelled_structures/FF-F2XEntry-G06a_1-pandda-model.pdb
G07	b	Trx2	xdsapp	dimple	10.17879/69998577695	FF-PanDDA-Daten2/xdsapp_dimple/pandda/processed_datasets/FF-F2XEntry-G07b_1/modelled_structures/FF-F2XEntry-G07b_1-pandda-model.pdb
G08	b	Dimer	xdsapp	dimple	10.17879/69998577695	FF-PanDDA-Daten2/xdsapp_dimple/pandda/processed_datasets/FF-F2XEntry-G08b_1/modelled_structures/FF-F2XEntry-G08b_1-pandda-model.pdb
H04	a	Dimer	frag	plex	10.17879/69998577695	FF-PanDDA-Daten2/frag_plex/pandda/processed_datasets/FF-F2XEntry-H04a_1/modelled_structures/FF-F2XEntry-H04a_1-pandda-model.pdb

H07	a	NADPH	xdsapp	dimple	10.17879/69998577695	FF-PanDDA-Daten2/xdsapp_dimple/pandda/processed_datasets/FF-F2XEntry-H07a_1/modelled_structures/FF-F2XEntry-H07a_1-pandda-model.pdb
H08	a	Dimer	xdsapp	dimple	10.17879/69998577695	FF-PanDDA-Daten2/xdsapp_dimple/pandda/processed_datasets/FF-F2XEntry-H08a_1/modelled_structures/FF-F2XEntry-H08a_1-pandda-model.pdb
H10	a	Dimer	frag	plex	10.17879/69998577695	FF-PanDDA-Daten2/frag_plex/pandda/processed_datasets/FF-F2XEntry-H10a_1/modelled_structures/FF-F2XEntry-H10a_1-pandda-model.pdb
H12	a	Dimer	frag	plex	10.17879/69998577695	FF-PanDDA-Daten2/frag_plex/pandda/processed_datasets/FF-F2XEntry-H12a_1/modelled_structures/FF-F2XEntry-H12a_1-pandda-model.pdb
